# Effects of ethanolic extracts of *Quercus*, *Cirsium vulgare*, and *Falcaria vulgaris* on gastric ulcer, antioxidant and inflammatory indices, and gene expression in rats

**DOI:** 10.14814/phy2.14954

**Published:** 2021-08-17

**Authors:** Ali Mohammad Basatinya, Javad Sajedianfard, Saeed Nazifi, Saied Hosseinzadeh, Mahbobeh Kamrani Mehni, Abolfazl Farahi, Kaveh Rahimi, Amin Derakhshanfar, Sina Salavati

**Affiliations:** ^1^ Department of Basic Sciences School of Veterinary Science Shiraz University Shiraz Iran; ^2^ Department of Clinical Science School of Veterinary Science Shiraz University Shiraz Iran; ^3^ Department of Hygiene and Food Quality Control School of Veterinary Science Shiraz University Shiraz Iran; ^4^ Diagnostic Laboratory Sciences and Technology Research Center School of Paramedical Sciences Shiraz University of Medical Sciences Shiraz Iran

**Keywords:** gastric ulcer, gene, inflammatory factors, oxidative enzymes, *Quercus brantii*

## Abstract

**Introduction:**

Gastric ulcer is a multifaceted process and is usually caused by mucosal damage. Herbal medicines have received much attention considering the side effects of chemical drugs. Nowadays, the use of herbal medicines has received much attention considering the side effects of chemical drugs. *Quercus brantii* Lindl, *Cirsium* *vulgare* (Savi) Ten, and *Falcaria* *vulgaris* Bernh are plants used as traditional phytomedicine for gastric ulcer diseases.

**Aim of the study:**

This study was aimed to investigate the protective effects of hydroalcoholic extracts of these herbs on ethanol‐induced gastric ulceration, in addition, to investigate the antioxidant, anti‐inflammatory, and gene expression.

**Materials and Methods:**

Thirty Sprague Dawley rats, (200–250 g), were divided into six groups: Control: intact animals; sham: gavaged with distilled water (14 days); negative control: gavaged with 20 mg/kg of omeprazole (14 days); experimental groups I, II, and III: gavaged with 500 mg/kg of the extract of *Falcaria* *vulgaris*, *Quercus brantii*, and *Cirsium* *vulgare*, respectively, (14 days). The number of ulcers and pathological parameters were assessed. The serum superoxide dismutase, catalase, glutathione peroxidase, malondialdehyde, total antioxidant capacity, albumin, total protein, haptoglobin, alpha‐1‐acid glycoprotein, total globulin, alpha‐2‐macroglobulin, C‐fos, C‐myc, and Caspase‐9 were measured by ELISA and RT‐PCR.

**Results:**

The extracts significantly reduced gastric ulcer (52.33%). The results showed that the *Quercus brantii* extract was more effective. There were significant differences between the serum levels of alpha‐1‐acid glycoprotein and those of alpha‐2‐macroglobulin. Also, there was a significant difference in the serum level of antioxidant parameters. Changes in the expression of the genes also confirmed the results suggested by other parameters. The expression levels of *C*‐*fos*, *C*‐*myc*, and *caspase*‐*9* were decreased, but the Bcl‐2 expression increased.

**Conclusion:**

The hydro‐alcoholic extracts revealed various protection and noticeable change in the expression of *caspase*‐*9*, *C*‐*myc*, *C*‐*fos*, and *Bcl*‐*2* genes in rats.

## Graphical abstract

The effect of ethanolic extracts of *Quercus* *brantii*, *Cirsium* *vulgare*, *and* *Falcaria* *vulgaris* on the prevention of ethanol‐induced gastric ulceration, the antioxidant and inflammatory factors, and expression of caspase‐9, C‐myc, C‐fos, and Bcl‐2 in rats demonstrated that the hydro‐alcoholic extracts of *Quercus* *brantii*, *Falcaria* *vulgaris* *and* *Cirsium* *vulgare* revealed various protection.

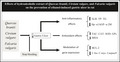

## 1 INTRODUCTION

Gastric ulcers refer to open sores, which occur in the gastric mucosa due to the inefficiency of the gastrointestinal defense system against damaging agents. Mucosal ulcers occur when there is an imbalance between the invading factors (acid, pepsin, bile) and gastrointestinal mucosal defense mechanisms, such as mucus and bicarbonate secretion, prostaglandins, and blood flow (Mota et al., 2008). Although tremendous progress has been made in inhibiting or reducing acid secretion and reinforcing the gastric mucosal barrier against the causes of gastric ulcer, the prevalence of this disease is still high. The use of synthetic drugs is usually associated with many adverse side effects, which has led to increased demand for medicinal plants. The most effective herbal compounds with potential anti‐ulcer properties include flavonoids, tannins, triterpenoids, and fatty acids, many of which can be found in *Quercus* *brantii* Lindl, *Cirsium* *vulgare* (Savi) Ten, and *Falcaria* *vulgaris* Bernh (Haghi & Hatami, 2010; Warren & Marshall, 1983). In Iranian Traditional Medicine, *Quercus* *brantii,* *Falcaria* *vulgaris*, and *Cirsium* *vulgare* have been used for angina throat, typical blood hemorrhoids, relieves stomach pain and its gas, strengthen the stomach, accelerate would healing, and antioxidant activities (Bahmani et al., 2015 and Goorani et al., 2019 and Sabudak et al., 2017). Gastric microcirculatory disturbance is considered to be the most important pathogenic process in ethanol‐induced gastric lesions, which is independent of gastric acid secretion (Oates & Hakkinen, 1988). Mechanisms involved in ethanol‐induced gastric ulcer (EIGU) include free radical production, inhibition of cell proliferation, and inflammatory cell accumulation. The cell has complex antioxidant, enzymatic, and non‐enzymatic systems which maintain the redox homeostasis state (Senthil et al., 2004).

*C*‐*fos* is a well‐known proto‐oncogene (Chiu et al., 1988). The expression of the *C*‐*fos* and *C*‐*myc* genes begins the ulcer healing process; these genes are usually called rapid‐ or acute‐phase genes. C‐fos is involved in various important cellular events, including proliferation and differentiation (Wong et al., 2000). In addition, *C*‐*fos* and *C*‐*myc* are involved in the mechanism of polyamine‐stimulated ulcer healing in stress‐related gastric mucosal ulcers (Gearhart et al., 2007).

The expression of many genes, as well as a number of coding peptides, increases after mucosal damage in the gastrointestinal mucosa. All products of these genes play an important compensatory role in healing the damaged mucosa (Wong et al., 2000).

Caspase‐9 has been reported as a major cause of apoptosis. The microarray results showed that caspase‐9 suppression led to significant overexpression of genes responsible for proliferation, growth, development, and cell division (Van Ba & Hwang, 2014).

Bcl‐2 and related cytoplasmic proteins are key regulators of apoptosis, tissue homeostasis, and protection against pathogens. Those that are more like Bcl‐2 participate in cell survival by inhibiting the adapters required to activate the proteases (caspases) that kill cells (Adams & Cory, 1998).

The aim of the present research was to investigate the effects of the ethanolic extracts of *Falcaria* *vulgaris*, *Quercus* *brantii*, and *Cirsium* *vulgare* on the serum SOD, CAT, GPx, MDA, TAC, ALB, TP, Hp, α**_1_**AGp, TG, α2M, as well as the expression of C‐myc, C‐fos, Bcl‐2, caspase‐9 mRNA, and histopathology of gastric tissue in gastric ulcer caused by EIGU.

## 2 MATERIALS AND METHODS

### 2.1 Laboratory Animals

Thirty adult Sprague Dawley rats, weighing 200–250 g, were randomly divided into six groups as follows: Control group: did not receive any special treatment throughout the study; sham group: distilled water was gavaged for 14 days; negative control group: Omeprazole (20 mg/kg) was gavaged for 14 days; experimental group I: ethanolic extract of *Falcaria* *vulgaris* (500 mg/kg) was gavaged for 14 days; experimental group II: ethanolic extract of *Quercus* *brantii* (500 mg/kg) was gavaged for 14 days; experimental group III: ethanolic extract of *Cirsium* *vulgare* (500 mg/kg) was gavaged for 14 days. At the end of the gavage period, all groups, except the control group, had no access to food for 24 h, and the EIGU was induced in the other groups using 1 ml/200 kg ethanol. The animals were anesthetized an hour after the induction of the ulceration with CO_2_ gas and blood was taken from the heart. Afterward, all rats were killed and their stomach was removed to evaluate the expression of the above‐mentioned genes, calculation of the number of ulcers, and examination of the pathological parameters of ulcers.

### 2.2 Serum‐related factors

Total protein and albumin were measured using commercial kits (Pars Azmoon) and alpha‐classical biochemical autoanalyzer apparatus manufactured by Sanjesh Co. Serum total globulin concentration was obtained by subtracting total protein and serum albumin. Serum haptoglobin and alpha‐1‐acid glycoprotein levels were measured by the sandwich ELISA method and commercial rat kits manufactured by Shanghai Crystal Day Biotech Co. Serum alpha‐2‐macroglobulin level was measured using the sandwich ELISA method and a rat‐specific commercial kit manufactured by Abcam Co.

The ZellBio GmbH kit was used to measure the activity of the SOD enzyme based on the colorimetric method at a wavelength of 420 nm. The quantitative measurement of GPx activity was carried out using the ZellBio kit based on the colorimetric method at 412 nm, as was recommended by the manufacturers. The catalase level, at an absorbance of 410 nm, was read by a spectrophotometer.

The lipid peroxidation (MDA) in the blood serum was evaluated by the ELISA kit (Cusabio Biotech Com). In this study, total antioxidant status was measured using a kit manufactured by ZellBio Co.

### 2.3 Quantitative real‐time PCR

The RT‐qPCR method was used to assess the expression of caspase‐9, C‐myc, C‐fos, and Bcl‐2 mRNA. The gastric tissue was isolated and gastric antrum was placed at −70℃ for RNA extraction. RNX‐Plus solution for the total RNA isolation (Sinaclon) was used for the extraction of RNA from each sample. The concentration of the extracted RNA was determined by the NanoDrop One C device (Thermo Scientific Co.). The amount of RNA in the samples was normalized with DEPC water. Easy™ cDNA Synthesis kit (Pars Tus Inc.) was used for cDNA synthesis. The expression of the plc mRNA in each sample was quantified by RT‐qPCR. 16S rRNA was used as the reference gene. The RT‐qPCR mixture contained 12.5 ml of SYBR® Green Real‐Time PCR master mix (Pars Tus Inc.), 0.8 ml of cDNA, 9.7 ml of DNase free water, and 1 ml for each forward and reverse primers (Table 1). RT‐qPCR reaction was completed by LightCycler device (Roche). The RT‐qPCR conditions were as follows: 94℃ for 5 min as initial denaturation followed by 40 cycles of 95℃ for 30 s, 56℃ for 30 s, and 72℃ for 30 s.

**TABLE 1:** Gene primers used for RT‐qPCR analysis

Primer	Primer Sequence	Gene
**F** **R**	ATGATGTTCTCGGGCTTCAACGCAGCAG AACCAATTCTTACTATGGCAAGCG	C‐fos
**F** **R**	AAACACAAACTTGAACAGCTAC ATTTGAGGCAGTTTACATTATGG	C‐myc
**F** **R**	AGTTCCCGGGTGCTGTCTAT GCCATGGTGTTTCTGCTCAC	Caspase−9
**F** **R**	GTGGTGGAGGAACTCTTCAG GTTCCACAAAGGCATCCCAG	Bcl−2
**F** **R**	ACCTTGGAAATAAATGGGAAG CTTCTGTGTTGCTGTAGTTGC	GAPDH

### 2.4 Histopathology

The microscopic study of the specimens was carried out as follows: (1) tissue fixation, (2) specimen transfer to cassettes, (3) tissue processing, (4) sectioning, (5) staining.

### 2.5 Statistical analysis

All the experiments were performed in triplicate. The results were reported as mean ± standard deviation. Differences at *p* < 0.05 were considered statistically significant. Statistical analysis of the results was performed using one‐way ANOVA and Tukey post hoc test. RT‐qPCR data were analyzed using Roche LightCycler 96.

## 3 RESULTS

### 3.1 Mean ulcer index

**IMAGE 1:**
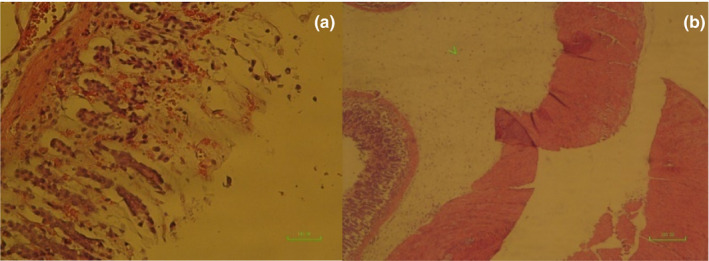
Pathology image of rat stomachs with EIGU after gavaged with distilled water, (second group). Epithelial layer is shortened with mild hemorrhage (a) (×200). Sever edema (arrowhead) of tunica submucosa (b) (×40)

**IMAGE 2:**
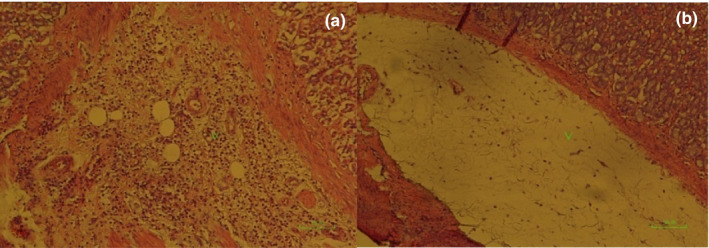
Pathology image of rat stomachs with EIGU after treatment with omeprazole (20 mg/kg), (third group). Mild mononuclear inflammatory cell infiltration in tunica submucosa (arrowhead) (x100) (a). Mild edema (arrowhead) of tunica submucosa (×100) (b)

**IMAGE 3:**
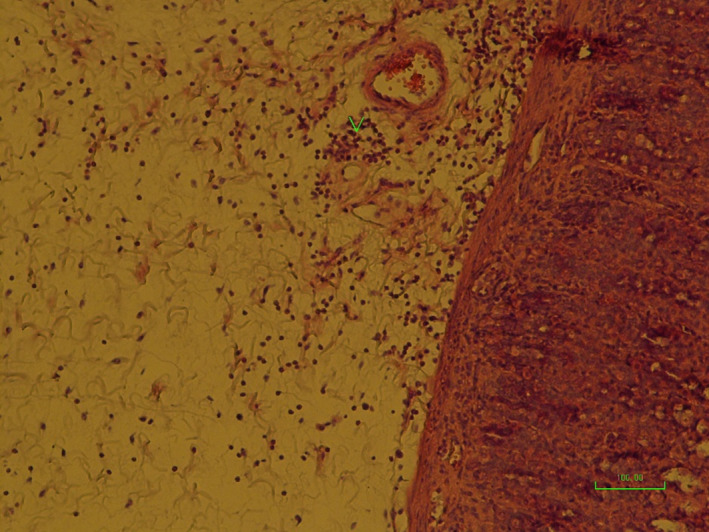
Pathology image of rat stomachs with EIGU after treatment with *Falcaria* *vulgaris* (500 mg/kg), (fourth group). Severe edema and mild mononuclear inflammatory cell infiltration of tunica submucosa (arrowhead) (×100)

**IMAGE 4:**
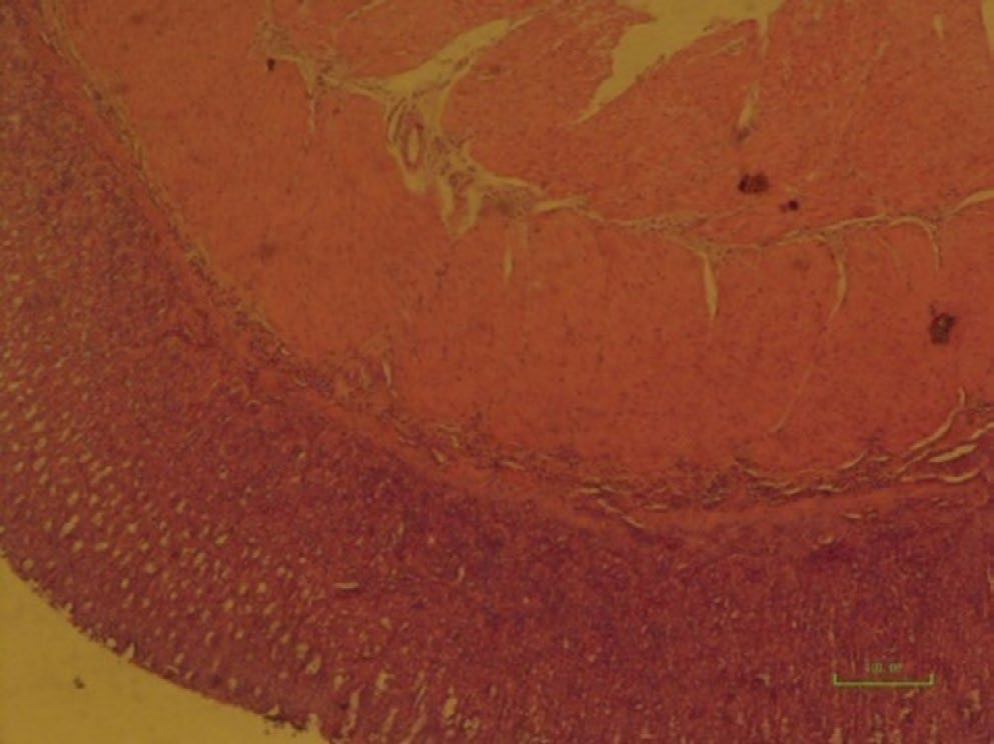
Pathology image of rat stomachs with EIGU after treatment with *Quercus* *brantii* (500 mg/kg), (fifth group). Normal microscopic structure of the gastric tissue (×40)

**IMAGE 5:**
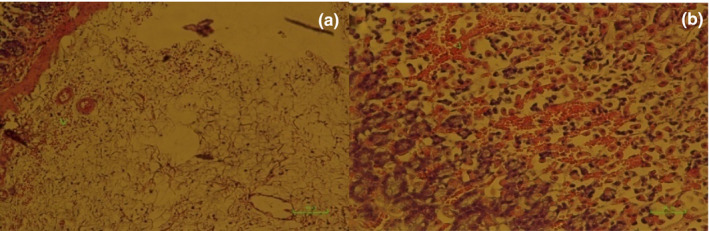
Pathology image of rat stomachs with EIGU after treatment with *Cirsium* *vulgare* (500 mg/kg) (the sixth group). Mild edema and hemorrhage of the tunica submucosa (arrowhead) (×100) (a). Mild hemorrhage (arrowhead) in the tunica submucosa (×200) (b)

**FIGURE 1:**
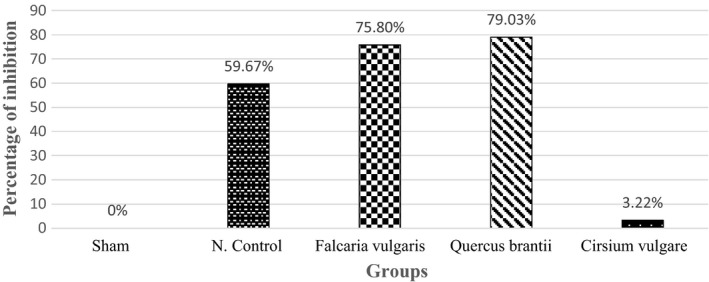
Effect of hydroalcoholic extract of *Quercus* *brantii* (500 mg/kg), *Cirsium* *vulgare* (500 mg/kg), and *Falcaria* *vulgaris* (500 mg/kg) on the percentage of gastric ulcer inhibition

The effect of ethanol on the mean ulcer index (MUI) is shown in Table 1. Ethanol in the sham rats caused significant mucosal damage with hemorrhagic necrotic lesions (Image 1). MUI decreased to 5.00 ± 0.70 and 3.00 ± 0.44 (mean ± SEM) in rats treated with omeprazole (Table 2, Image 2) and *Falcaria* *vulgaris* hydroalcoholic extract (Image 3), respectively. MUI also decreased significantly to 2.60 ± 1.07 (mean ± SEM) in rats treated with *Quercus* *brantii* hydroalcoholic extract (Image 4). MUI in rats treated with *Cirsium* *vulgare* hydroalcoholic extract (500 mg/kg) was measured as 12.0 ± 80.80 (mean ± SEM) (Image 5), which showed no significant difference compared to the sham group (Table 1). The ulcer inhibition rate of hydroalcoholic extracts of *Falcaria* *vulgaris*, *Quercus* *brantii*, and *Cirsium* *vulgare* was 75.80, 79.03, and 3.22%, respectively (Figure 1).

**TABLE 2:** Effect of the hydroalcoholic extract of *Quercus* *brantii* (500 mg/kg), *Cirsium* *vulgare* (500 mg/kg), and *Falcaria* *vulgaris* (500 mg/kg) on MUI, and ulcer inhibition rate in rats

Group	Mean Ulcer Index (MUI)	Percentage inhibition
Control	0.0	—
Sham	12.40 ± 0.74^a^	—
Negative Control	5.0 ± 0.70^b^	59.675%
*Falcaria* *Vulgaris*	3.0 ± 0.44^bc^	75.80%
*Quercus* *Brantii*	2.60 ± 1.07^c^	79.03%
*Cirsium* *Vulgare*	12.80 ± 0.80^a^	3.22%

The results are reported as the mean ± SEM. ANOVA followed by Tukey's test. *p* < 0.05. Different letters are used to indicate a significant difference in a column.

### 3.2 Antioxidant parameters

#### 3.2.1 TAC activity

**FIGURE 2:**
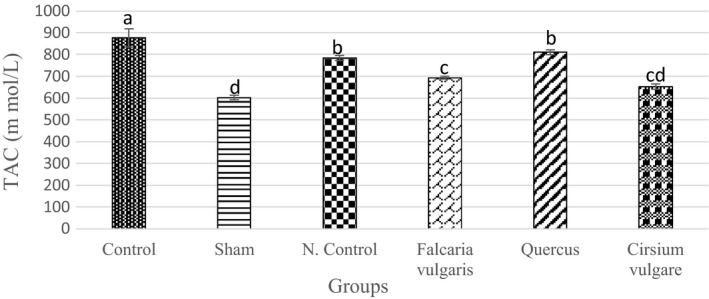
Effect of hydroalcoholic extract of *Quercus* *brantii* (500 mg/kg), *Cirsium* *vulgare* (500 mg/kg), and *Falcaria* *vulgaris* (500 mg/kg) on the serum TAC level. The results are reported as the mean ± SEM. ANOVA followed by Tukey's test. *p* ˂ 0.05. Different letters are used to indicate a significant difference between the groups

A significant decrease was observed in the TAC activity in the serum of sham‐treated rats compared to that of the control group (Table 3). The TAC level increased significantly in the *Quercus* *brantii*‐ and *Falcaria* *vulgaris*‐treated groups compared to that of the sham group (*p* < 0.05) (Figure 2).

**TABLE 3:** Effect of the hydroalcoholic extract of *Quercus* *brantii* (500 mg/kg), *Cirsium* *vulgare* (500 mg/kg), and *Falcaria* *vulgaris* (500 mg/kg) on antioxidant factors

Group	SOD (µ/ml)	GPx (µ/ml)	Catalase (µ/ml)	MDA (µmol/L)	TAC (mmol/L)
Control	95.06 ± 2.75^a^	118.08 ± 3.53^a^	542.61 ± 25.79^a^	19.62 ± 0.31^a^	875.95 ± 41.13^a^
Sham	60.84 ± 1.87^c^	69.88 ± 2.44^c^	326.3 ± 9.71^d^	22.06 ± 0.57^c^	602.04 ± 10^d^
Negative Control	87.06 ± 1.18^b^	99.38 ± 1.25^b^	408.16 ± 8.08^bc^	20.5 ± 0.19^ab^	783.94 ± 11.42^b^
*Falcaria* *Vulgaris*	73.88 ± 0.7^c^	84.55 ± 1.48^c^	370.96 ± 6.4^c^	21.24 ± 0.38^bc^	692.8 ± 7.06^c^
*Quercus* *Brantii*	89.42 ± 1.15^b^	105.36 ± 1.88b	426.86 ± 8.61^b^	20.18 ± 0.18^ab^	810.2 ± 10.89^b^
*Cirsium* *Vulgare*	68.6 ± 1.06^d^	77.36 ± 2.06^d^	327.56 ± 6.3^d^	21.7 ± 0.39^c^	652.04 ± 12.71 cd

The results are reported as the mean ± SEM. ANOVA followed by Tukey's test. *p* ˂ 0.05. Different letters are used to indicate a significant difference in a column

#### 3.2.2 SOD activity

**FIGURE 3:**
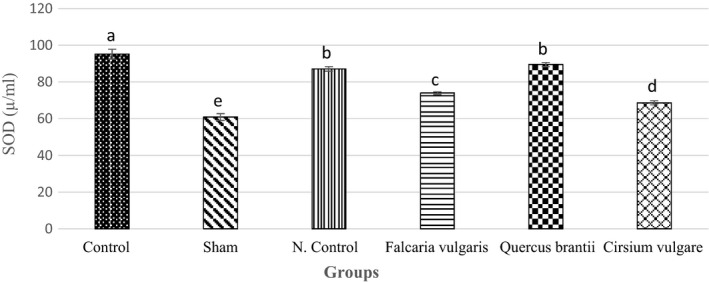
Effect of hydroalcoholic extract of *Quercus* *brantii* (500 mg/kg), *Cirsium* *vulgare* (500 mg/kg), and *Falcaria* *vulgaris* (500 mg/kg) on the serum SOD level. The results are reported as the mean ± SEM. ANOVA followed by Tukey's test. *p* ˂ 0.05. Different letters are used to indicate a significant difference between the groups

A significant decrease was observed in the SOD activity in the serum of the sham group compared to that of the controls (60.84 ± 1.87) (*p* < 0.05) (Table 3). Among the study groups, the group treated with *Quercus* *brantii* hydroalcoholic extract (89.42 ± 1.15) and omeprazole (87.06 ± 1.18) showed the most inhibitory effect on SOD activity (*p* < 0.05) (Figure 3).

#### 3.2.3 GPx activity

**FIGURE 4:**
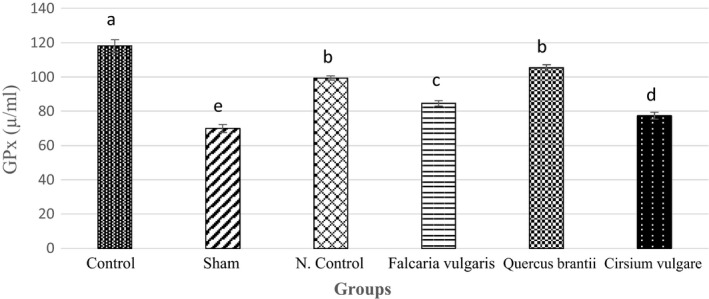
Effect of hydroalcoholic extract of *Quercus* *brantii* (500 mg/kg), *Cirsium* *vulgare* (500 mg/kg), and *Falcaria* *vulgaris* (500 mg/kg) on the serum GPx level. The results are reported as the mean ± SEM. ANOVA followed by Tukey's test. *p* ˂ 0.05. Different letters are used to indicate a significant difference between the groups

The results of different treatments on GPx activity are shown in Figure 4. The measured GPx levels were significantly different in all groups (*p* < 0.05). The results showed that the *Quercus* *brantii* extract‐treated group had a stronger inhibitory effect on GPx than the group treated with omeprazole (105.36 ± 1.88) (Figure 4, Table 3).

#### 3.2.4 CAT activity

**FIGURE 5:**
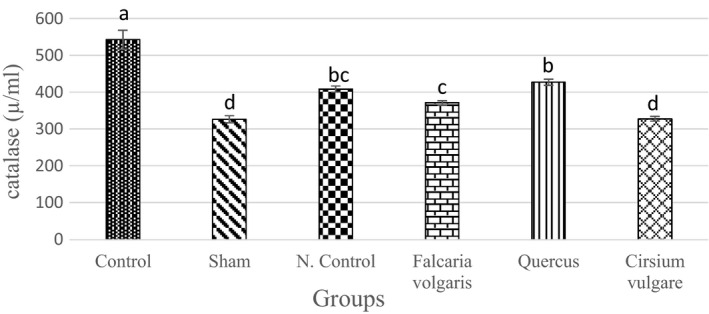
Effect of hydroalcoholic extract of *Quercus* *brantii* (500 mg/kg), *Cirsium* *vulgare* (500 mg/kg), and *Falcaria* *vulgaris* (500 mg/kg) on the serum CAT level. The results are reported as the mean ± SEM. ANOVA followed by Tukey's test. *p* ˂ 0.05. Different letters are used to indicate a significant difference between the groups

The results showed a statistically significant increase in the CAT levels in the negative control and experimental groups treated with *Quercus* *brantii* and *Falcaria* *vulgaris* extracts compared to those of the sham group (*p* < 0.05) (327.56 ± 6.3) (Figure 5 and Table 3).

#### 3.2.5 MDA activity

**FIGURE 6:**
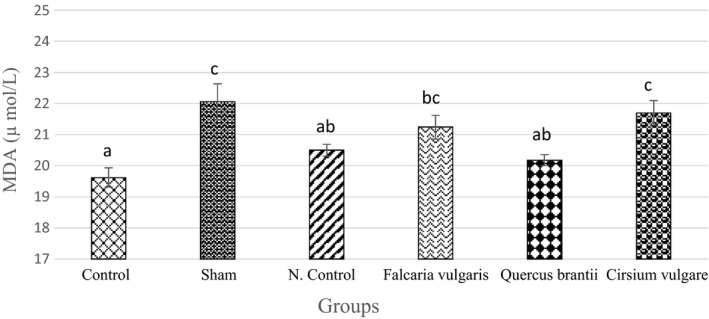
Effect of hydroalcoholic extract of *Quercus* *brantii* (500 mg/kg), *Cirsium* *vulgare* (500 mg/kg), and *Falcaria* *vulgaris* (500 mg/kg) on the serum MDA level. The results are reported as the mean ± SEM. ANOVA followed by Tukey's test. *P* ˂ 0.05. Different letters are used to indicate a significant difference between the groups

The results of MDA measurement showed that the MDA levels decreased significantly in rats receiving omeprazole and the hydroalcoholic extract of *Quercus* *brantii* and *Falcaria* *vulgaris* (*p* < 0.05). The *Quercus* *brantii* extract had the weakest inhibitory effect on MDA (20.18 ± 0.18) (Table 3 and Figure 6).

### 3.3 Inflammatory factors

#### 3.3.1 Albumins

**FIGURE 7:**
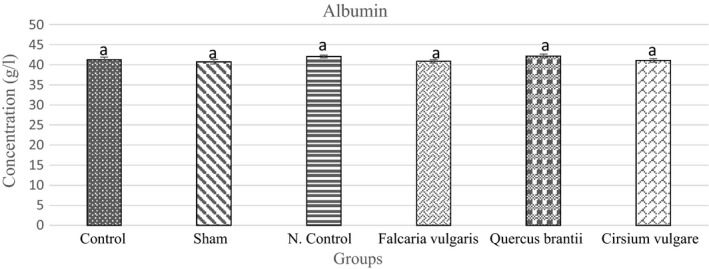
Effect of hydroalcoholic extract of *Quercus* *brantii* (500 mg/kg), *Cirsium* *vulgare* (500 mg/kg), and *Falcaria* *vulgaris* (500 mg/kg) on the serum albumin level. The results are reported as the mean ± SEM. ANOVA followed by Tukey's test. *p* ˂ 0.05. Different letters are used to indicate a significant difference between the groups

The results showed a reduced serum albumin level in the sham group as compared to that of the control group. Serum albumin levels increased in the negative control and treatment groups, but it was higher in the treatment group II, which received the hydroalcoholic extract of *Quercus* *brantii* (Figure 7, Table 4).

**TABLE 4:** Effect of the hydroalcoholic extract of *Quercus* *brantii* (500 mg/kg), *Cirsium* *vulgare* (500 mg/kg), and *Falcaria* *vulgaris* (500 mg/kg) on inflammatory factors

Group	Total protein (g/L)	Albumin (g/L)	Total globulin (g/L)	Haptoglobin (mg/ml)	α1‐Acid glycoprotein (µg/ml)	α2‐Macroglobulin (ng/ml)
Control	75.56 ± 0.76^a^	41.22 ± 0.66^a^	34.34 ± 1.24^a^	0.51 ± 0.03^a^	175.40 ± 3.66^a^	55.6 ± 2.56^a^
Sham	73.16 ± 1.48^a^	40.74 ± 0.58^a^	32.42 ± 1.50^a^	0.70 ± 0.04^b^	197.40 ± 5.91^b^	164.00 ± 8.06^d^
Negative Control	73.14 ± 1.55^a^	42.04 ± 0.37^a^	31.10 ± 1.83^a^	0.66 ± 0.04^b^	190.4 ± 4.44^ab^	78.80 ± 4.29^b^
*Falcaria* *Vulgaris*	73.46 ± 1.49^a^	40.84 ± 0.43^a^	32.62 ± 1.56^a^	0.68 ± 0.04^b^	193.80 ± 5.80^b^	144.40 ± 8.03^c^
*Quercus* *Brantii*	73.36 ± 1.55^a^	42.16 ± 0.51^a^	31.20 ± 1.55^a^	0.65 ± 0.04^b^	185.80 ± 4.61^ab^	70.80 ± 4.17^ab^
*Cirsium* *Vulgare*	73.38 ± 1.36^a^	41.06 ± 0.41^a^	32.32 ± 1.48^a^	0.69 ± 0.04^b^	195.40 ± 5.60^b^	153.60 ± 8.55 cd

The results are reported as the mean ± SEM. ANOVA followed by Tukey's test. *p* ˂ 0.05. Different letters are used to indicate a significant difference in a column

#### 3.3.2 Total protein

**FIGURE 8:**
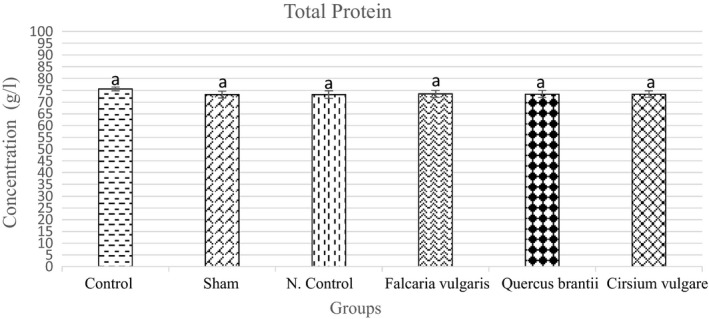
Effect of hydroalcoholic extract of *Quercus* *brantii* (500 mg/kg), *Cirsium* *vulgare* (500 mg/kg), and *Falcaria* *vulgaris* (500 mg/kg) on the serum total protein level. The results are reported as the mean ± SEM. ANOVA followed by Tukey's test. *p* ˂ 0.05. Different letters are used to indicate a significant difference between the groups

The serum total protein level was lower in the sham group than that of the control group. The serum total protein levels increased in all the treatment groups compared to those of the negative control group. Among the treatment groups, treatment group I (*Cirsium* *vulgare*) rendered better performance than other groups (Figure 8, Table 4).

#### 3.3.3 Haptoglobin

**FIGURE 9:**
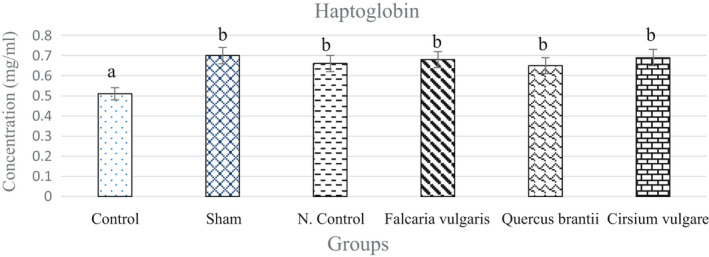
Effect of hydroalcoholic extract of *Quercus* *brantii* (500 mg/kg), *Cirsium* *vulgare* (500 mg/kg), and *Falcaria* *vulgaris* (500 mg/kg) on the serum haptoglobin level. The results are reported as the mean ± SEM. ANOVA followed by Tukey's test. *p* ˂ 0.05. Different letters are used to indicate a significant difference between the groups

There was a significant increase in the level of haptoglobin in all groups compared to that of the control (Figure 9) (*p* < 0.05). Among the treatment groups, treatment group II rendered better results than the negative control group and caused a decrease in the serum haptoglobin levels (Figure 9 and Table 4).

#### 3.3.4 Total globulin

**FIGURE 10:**
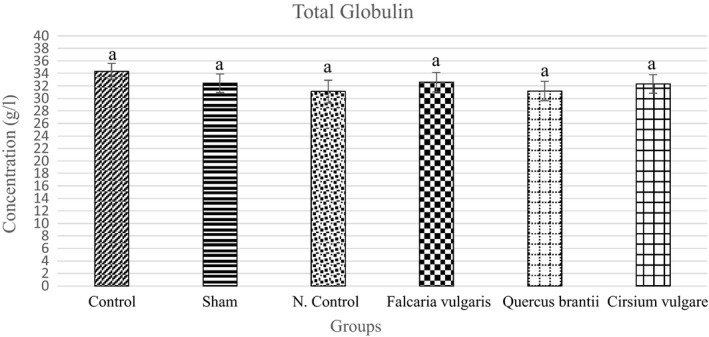
Effect of hydroalcoholic extract of *Quercus* *brantii* (500 mg/kg), *Cirsium* *vulgare* (500 mg/kg), and *Falcaria* *vulgaris* (500 mg/kg) on the serum total globulin level. The results are reported as the mean ± SEM. ANOVA followed by Tukey's test. *p* ˂ 0.05. Different letters are used to indicate a significant difference between the groups

The results showed a lower total globulin level in the sham group than that of the control group. This difference between these two groups showed that the total globulin decreased in rats with gastric ulcer and that the extracts used could increase the total globulin level. In the case of the total globulin levels, the treatment group I (*Cirsium* *vulgare*) showed more effective results (Figure 10 and Table 4).

#### 3.3.5 Alpha‐1‐acid glycoprotein

**FIGURE 11:**
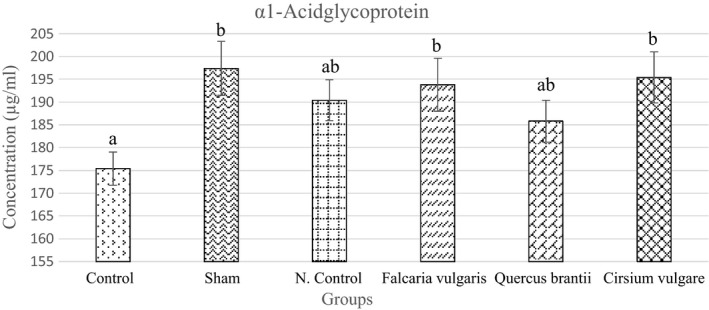
Effect of hydroalcoholic extract of *Quercus* *brantii* (500 mg/kg), *Cirsium* *vulgare* (500 mg/kg), and *Falcaria* *vulgaris* (500 mg/kg) on the serum total alpha‐1‐acid glycoprotein level. The results are reported as the mean ± SEM. ANOVA followed by Tukey's test. *p* ˂ 0. 05. Different letters are used to indicate a significant difference between the groups

The evaluation of the α1AGP level showed a significant increase in the sham group compared to the level observed in the control. Among the different treatment groups, treatment group II was more effective in reducing α1AGP levels (Figure 11 and Table 4).

#### 3.3.6 Alpha‐2‐macroglobulin

**FIGURE 12:**
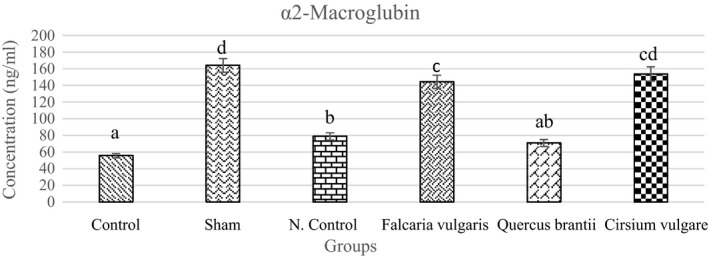
Effect of hydroalcoholic extract of *Quercus* *brantii* (500 mg/kg), *Cirsium* *vulgare* (500 mg/kg), and *Falcaria* *vulgaris* (500 mg/kg) on the serum total alpha‐2‐macroglobulin level. The results are reported as the mean ± SEM. ANOVA followed by Tukey's test. *p* ˂ 0.05. Different letters are used to indicate a significant difference between the groups

The results showed statistically different α‐2M levels between the groups (*p* < 0.05). There was a significant difference between the control and other groups, except the treatment group II (*Quercus* *brantii* extract) which rendered better results (Figure 12 and (Table 4).

### 3.4 Expression of caspase‐9, *C*‐*myc*, *C*‐*fos*, and *Bcl*‐*2* genes

#### 3.4.1 *C*‐*fos*

**FIGURE 13:**
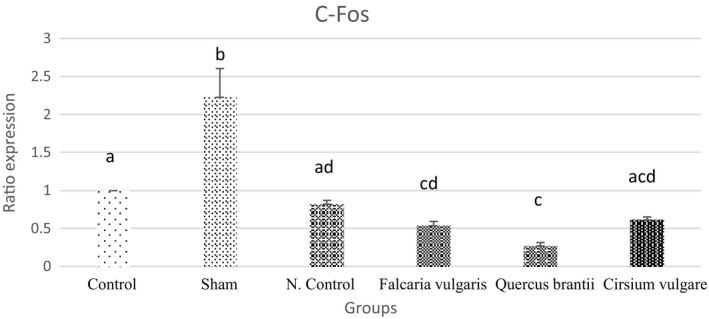
Effect of hydroalcoholic extract of *Quercus* *brantii* (500 mg/kg), *Cirsium* *vulgare* (500 mg/kg), and *Falcaria* *vulgaris* (500 mg/kg) on the C‐fos expression level. The results are reported as the mean ± SEM. ANOVA followed by Tukey's test. *p* ˂ 0.05. Different letters are used to indicate a significant difference between the groups

C‐fos expression is increased in gastric ulcer disease. The results showed a reduction in the C‐fos expression after the consumption of the extracts by the rats. Among the treatment groups, the treatment group II (*Quercus* *brantii* extract) showed better results, even better than the omeprazole‐treated group, and caused a further reduction in the C‐fos expression (Figure 13 and Table 5).

**TABLE 5:** Effect of the hydroalcoholic extract of *Quercus* *brantii* (500 mg/kg), *Cirsium* *vulgare* (500 mg/kg), and *Falcaria* *vulgaris* (500 mg/kg) on expression values of the measured genes

Group	C‐myc (R.E.)	C‐fos (R.E.)	Caspase‐9 (R.E.)	BCL2 (R.E.)
Control	1	1	1	1
Sham	4.346	2.224	3.434	0.301
Negative Control	0.569	0.819	0.888	2.279
*Falcaria* *Vulgaris*	0.477	0.535	0.650	3.605
*Quercus* *Brantii*	0.339	0.269	0.435	5.133
*Cirsium* *Vulgare*	0.378	0.614	0.489	1.905

#### 3.4.2 *C*‐*myc*

**FIGURE 14:**
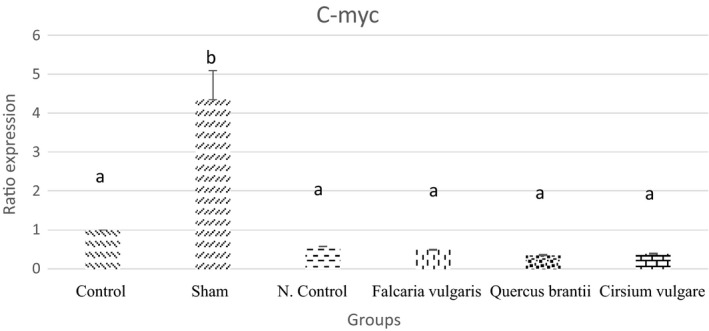
Effect of hydroalcoholic extract of *Quercus* *brantii* (500 mg/kg), *Cirsium* *vulgare* (500 mg/kg), and *Falcaria* *vulgaris* (500 mg/kg) on the C‐myc expression level. The results are reported as the mean ± SEM. ANOVA followed by Tukey's test. *p* ˂ 0.05. Different letters are used to indicate a significant difference between the groups

The elevated expression of the c‐myc gene was found in the gastric ulcer due to the mucosal damage. The results showed a reduced c‐myc level after the consumption of hydroalcoholic extracts of *Falcaria* *vulgaris*, *Quercus* *brantii*, and *Cirsium* *vulgare* herbs. Among the treatment groups, only the treatment group II (*Quercus* *brantii* extract) was able to reduce the expression of the above‐mentioned gene (Figure 14 and Table 5).

#### 3.4.3 *Caspase*‐*9*

**FIGURE 15:**
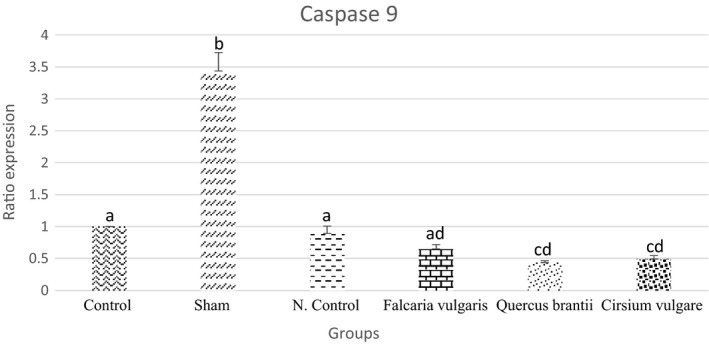
Effect of hydroalcoholic extract of *Quercus* *brantii* (500 mg/kg), *Cirsium* *vulgare* (500 mg/kg), and *Falcaria* *vulgaris* (500 mg/kg) on the caspase‐9 expression level. The results are reported as the mean ± SEM. ANOVA followed by Tukey's test. *p* ˂ 0.05. Different letters are used to indicate a significant difference between the groups

The caspase‐9 expression is increased in gastric ulcer disease. The results showed a reduction in the caspase‐9 expression after the consumption of the extracts. The treatment group II, treated with the *Quercus* *brantii* extract, rendered better results, even better than the omeprazole‐treated group, and caused a further reduction in the expression of the above‐mentioned gene (Figure 15 and Table 5).

#### 3.4.4 *Bcl*‐*2*

**FIGURE 16:**
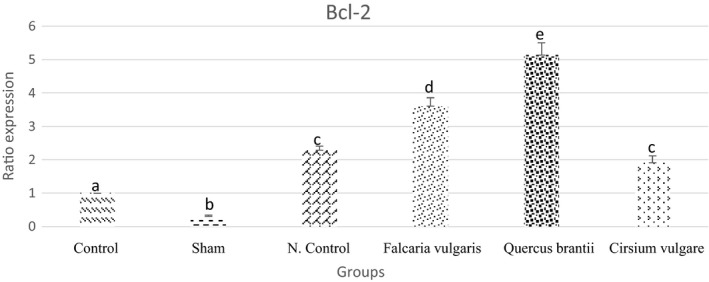
Effect of hydroalcoholic extract of *Quercus* *brantii* (500 mg/kg), *Cirsium* *vulgare* (500 mg/kg), and *Falcaria* *vulgaris* (500 mg/kg) on the Bcl‐2 expression level. The results are reported as the mean ± SEM. ANOVA followed by Tukey's test. *p* ˂ 0.05. Different letters are used to indicate a significant difference between groups

Bcl‐2 expression was reduced in response to gastric ulcer disease. The results showed an elevated Bcl‐2 expression after the consumption of the extracts of the herbs. Among the treatment groups, the treatment group II, which consumed the hydroalcoholic *Quercus* *brantii* extract, showed better results than the omeprazole‐treated group and increased Bcl‐2 expression (Figure 16 and Table 5).

## 4 DISCUSSION

Gastric ulcer is caused by an imbalance between the invasive agents and protective mechanisms of the gastric mucosa (Baiu & Azagury, 2019; Demir et al., 2003). Ethanol increases oxidative stress and causes changes in the levels of calcium secreted in the gastric cells that may cause gastric mucosal damage (Wong et al., 1991).

In the present study, lesions were observed in the gastric tissue sections of the sham group (Table 1). The results showed that the extracts of *Quercus* *brantii*, *Falcaria* *vulgaris*, and *Cirsium* *vulgare*, respectively, had the highest effect in relieving EIGU due to their antioxidant and anti‐inflammatory properties, as has been proved for other herbs in previous studies (Zainulddin et al., 2016). The results showed that the hydroalcoholic extract of *Quercus* *brantii* showed therapeutic and protective effects on EIGU because the tissue structure was very close to the normal structure after the treatment and no edema and inflammatory cell infiltration were observed in the tissue sections (Image 4).

Also, the experimental treatment group II (*Quercus* *brantii* extract) showed significant antioxidant activity by decreasing the MDA level and increasing the CAT, SOD, GPx, and TAC levels in response to the alcohol‐induced oxidative stress. The gastric protective activity of this *Quercus* *brantii* extract may be due to its antioxidant property. The results showed that the protective effects of tannin and *Quercus* *brantii* phenolic compounds in the gastrointestinal tract were due to their antioxidant properties (Khennouf et al., 1999).

These inhibitory effects of these herbs on the antioxidant enzymes are further evidence of the presence of compounds with antioxidant properties in them. Other researchers have also confirmed that the antioxidant status can be improved by other herbs (Kath & Gupta, 2006; Zainulddin et al., 2016).

GPx could effectively reduce hydrogen peroxide and lipid peroxide to water and lipid alcohols, respectively (Yao et al., 2006). In the present study, elevated GPx levels were observed in all three experimental groups compared to those of the sham group. *Quercus* *brantii* extract represented a greater inhibitory effect on GPx, probably due to its potential antioxidant activity. By removing hydrogen peroxide, catalase indirectly neutralizes superoxide radicals, which are converted to hydrogen peroxide by SOD (Turner & Lysiak, 2008).

In this study, the highest TAC level was obtained in the experimental groups, which were treated with *Quercus* *brantii*, *Falcaria* *vulgaris*, and *Cirsium* *vulgare* extracts, respectively. There are reports showing that phenolic compounds, including flavonoid compounds in plant extracts, have protective effects on the gastric and intestinal mucus (de Lira Mota et al., 2009; Zakaria et al., 2014). Thus, these compounds, due to their antioxidant effects, can be helpful in healing ulcers by eliminating free radicals (Lewis & Shaw, 2001). Flavonoids heal deep necrotic ulcers and prevent the large shedding of epithelial cells (Hosseinzadeh et al., 2002). Previous research has shown that flavonoids can increase the rate of gastric ulcer healing by increasing the synthesis of endogenous prostaglandins, inhibiting the secretion of histamine, and eliminating *Helicobacter* *pylori* and anti‐acid secretory agents (Olaleye & Farombi, 2006).

Albumin synthesis is inhibited by inflammatory mediators, including IL‐6 (Caraceni et al., 2013). In response to the acute inflammatory phase, it also reduces hepatic albumin synthesis and mRNA levels (Kulkarni et al., 1985). High levels of albumin excretion from the vessel wall can cause a decrease in the serum albumin in the inflammatory disease (Oates & Hakkinen, 1988). The fact that serum albumin is an acute‐phase negative protein implies that serum albumin concentration is a good marker for inflammation (Don & Kaysen, 2004). The results revealed that all three extracts were able to increase serum albumin levels.

The herbs used in this study contained anti‐inflammatory compounds, such as flavonoids (Alibabaei et al., 2010). Flavonoids are considered nitric oxide synthase (NOS) inhibitors, thereby exerting their anti‐inflammatory effects by reducing NO and prostaglandins (Hoodgar et al., 2011). Flavonoids, such as apigenin, reduce the accumulation of floating lipids; thus, they can reduce inflammation by inhibiting the accumulation of receptors and signal cascade (Nasri et al., 2009). Flavonoids also regulate blood vessel permeability (Di Carlo et al., 1999).

All the three extracts used in this study were able to increase the serum albumin levels (especially *Quercus* *brantii*). The elevated serum albumin level was due to the flavonoid‐induced inhibition of inflammatory mediators which were present in the herbs. These inflammatory mediators inhibit the synthesis of albumin and decrease its serum levels in some diseases, such as gastric ulcer (Di Carlo et al., 1999; Jung et al., 2012; Martínez‐Vázquez et al., 1998).

The total serum protein level in the sham group decreased compared to that of the control group. The results obtained from the treatment groups showed that the hydroalcoholic extracts of *Cirsium* *vulgare*, *Quercus* *brantii*, and *Falcaria* *vulgaris* were able to bring the serum total protein its normal level in the control group. The decrease in the serum total protein level in response to gastric ulcers can be attributed to the increased permeability of the vessels due to inflammation (Ballmer et al., 1992; Caraceni et al., 2013; Don & Kaysen, 2004).

The serum haptoglobin levels increased in the sham group compared to those of the control group, and all three extracts could reduce its levels and bring it to its normal levels. Among the three herbal extracts used, *Quercus* *brantii* extract was more effective. The inflammatory response at the site of the ulcers is the main stimulus that increases the haptoglobin synthesis rate in the gastric ulcer (Walker et al., 1990).

Haptoglobin is an acute‐phase α2‐glycoprotein (Javid, 1978). It has an important anti‐inflammatory function in the body (Sadrzadeh & Bozorgmehr, 2004). Serum haptoglobin levels have been identified as an index for tissue damage (Bilgrami et al., 1980). Haptoglobin level is increased in response to stress, acute inflammation, or necrosis of the tissue, probably due to the stimulation of the synthesis of haptoglobins (Walker et al., 1990).

The common hypothesis is that haptoglobin possesses an anti‐inflammatory activity, which means that haptoglobin reduces the inflammatory process by, for example, producing IL‐6, which itself determines the rate and duration of the expression of acute‐phase protein haptoglobin (Wang et al., 2001). According to the results, it can be stated that it was the flavonoids in the studied herbs that decreased the serum haptoglobin levels in rats. The decrease in the haptoglobin levels caused by the flavonoids could be attributed to their good anti‐inflammatory activity, which is mainly due to the inhibition of the production of IL‐6, a major stimulus for the expression of the haptoglobin gene.

The results indicate a reduction in the total globulin in the inflammatory process. The plant extracts in this study could raise the serum total globulin levels, and bring it closer to its normal range, and be even more effective than omeprazole. Meanwhile, the *Falcaria* *vulgaris* extract had a better effect than the other two extracts (even omeprazole). The results showed that the extracts were able to suppress gastric ulcer inflammation and increase the globulin synthesis rate (Fahey & Robinson, 1963).

The main reason for the decrease in the serum total globulin levels in the peptic ulcer is that the inflammatory reaction inhibits the synthesis of gamma globulin, which is the major globulin. Therefore, if the inhibitory factors can be stopped, the synthesis of globulins, especially gamma globulins, will return to their normal state.

In the case of α‐1AGP, the results indicated that this acute‐phase protein was overexpressed in gastric ulcer disease. The extracts used in this study significantly reduced the α‐1AGP level. The *Quercus* *brantii* extract had the best effect and significantly reduced the α‐1AGP level. The effect of the *Quercus* *brantii* extract was even better than that of the omeprazole. Inflammatory mediators stimulate α‐1AGP synthesis. Α‐1AGP is one of the most important acute‐phase proteins in humans and rats, and its serum concentration increases in response to systemic tissue damage, inflammation, or infection (Fournier et al., 2000). Α‐1AGP is a natural anti‐inflammation agent (Murata et al., 2004).

The results revealed that a reduced α‐1AGP level could be due to the following mechanisms. Cytokines are one of the regulatory factors for α‐1AGP. Flavonoids are a potent antioxidant and anti‐inflammatory compound that are able to inhibit histamine and some cytokines released from various cells (Mitchell et al., 1997). Therefore, it can be stated that a decrease in the serum α‐1AGP level is due to the inhibition of cytokines. When IL‐6 is inhibited, one of the regulators of α‐1AGP disappears and this, in turn, reduces its serum levels.

Alpha‐2‐macroglobulin (α‐2M) is an important acute‐phase protein. The level of this factor increased in the groups in which gastric ulcer was induced, an increase which could be attributed to the inflammation in the peptic ulcer. The studied extracts could significantly reduce the α‐2M levels and bring them closer to the normal levels.

Α‐2M levels increase when serum albumin levels are low (Stevenson et al., 1998). As a result, if the serum albumin level increases, the serum α‐2M level will decrease (Caraceni et al., 2013). As reported under the albumin section, the serum albumin level decreased in this study. In short, it can be stated that inflammation caused by peptic ulcer can increase the serum levels of proteinase inhibitors and anti‐inflammatory substances, such as α‐2M.

Α 2M is the major acute‐phase protein (marker) in rats. This protein can show marked changes, meaning that it is significantly increased after ulceration. *Quercus* *brantii* can bring α‐2M level closer to its normal level. The results showed that α‐2M was a very good marker for the overall evaluation of the effects of the extracts used in this study, a finding which is consistent with the results obtained from macroscopic and pathological studies.

In this study, the results of the gene expression assessment showed that *C*‐*fos* and *C*‐*myc* proto‐oncogenes, which are involved in the control of the proliferation of different cell types, were overexpressed after the induction of gastric mucosal damage. After being gavaged, the hydroalcoholic extracts of *Cirsium* *vulgare*, *Quercus* *brantii*, and *Falcaria* *vulgaris*, especially *Quercus* *brantii*, could successfully reduce the expression of the above‐mentioned genes with regard to their antioxidant, anti‐inflammatory, and thus protective properties. These results indicate that the studied extracts could regulate the expression of *C*‐*fos* and *C*‐*myc* and have prophylactic effects. The expression of *caspase*‐*9*, an apoptosis‐inducing factor, and *Bcl*‐*2* gene, an antiapoptotic factor, decreased and increased, respectively, in all the three treatment groups after the consumption of the hydroalcoholic extracts. Botanical compounds with antioxidant activity include flavonoids, saponins, and tannins. Several mechanisms, including an increase in the cutaneous prostaglandins and a decrease in the secretion of mast cells by the inhibition of both histidine decarboxylase and *H*. *pylori*, have been proposed to account for the gastric protective effects of flavonoids. In addition, flavonoids are known as factors killing free radicals. Quercetin is the most common subgroup of flavonoid molecules. It has been reported that quercetin can prevent ethanol‐induced gastric mucosal lesions and increase the levels of neutral glycoproteins in the gastric mucosa. Given that these proteins are the most abundant and, perhaps, the most important proteins in the gastric mucosa, it can be assumed that their quantitative replacement means that the gastric mucosa tissue returns to its normal state and that this, in turn, improves the protective capacity of the body against ethanol‐induced damage (Borrelli & Izzo, 2000).

The results of previous studies on EIGU in rats have shown that the hydroalcoholic extract of *Falcaria* *vulgaris* *can* significantly reduce the gastric ulcer index at all doses. This extract contains tannins and flavonoids, and these compounds have high antioxidant content, which is the main reason for the protective effects of this extract on the gastrointestinal tract (Khazaei & Salehi, 2006; Pourmorad et al., 2006).

Decreased *C*‐*fos* and *C*‐*myc* expression could be due to the large amounts of phenolic compounds in the *Quercus* *brantii* that contain high antioxidant potential. Extracts isolated from the *Quercus* *brantii* seed and skin have biological and medicinal properties, such as antibacterial and ulcer healing effects. The *Quercus* *brantii* extracts can downregulate proto‐oncogenes, an understanding that can justify the results of the present study. The results showed that three extracts used in the treatment groups reduced the *C*‐*myc* expression level.

*Caspase*‐*9* expression significantly decreased in the treatment groups. Each of the extracts reduced the expression of caspase‐9 because of their antioxidant and anti‐inflammatory properties. H. pylori infection can induce apoptosis in gastric epithelial cells (Moss et al., 1996) which can, subsequently, increase the cellular effect in response to apoptosis (Kroemer, 1997). Cell proliferation and apoptosis are among the essential events in the gastric tissue cell turnover, and any change in this balance can be the biological basis for gastric carcinogenesis (Maeda et al., 2002). There is evidence that apoptosis is involved in the pathogenesis of ulcer formation and healing. Apoptosis can be caused by a variety of internal and external stimuli, such as nutrient deprivation, mechanical stimuli, and stress (Rai et al., 2005). It can be said that *caspase*‐*9* was downregulated in this study due to the protective effects of the extracts in question and the use of these extracts to reduce tissue damage.

In this study, the extracts of *Cirsium* *vulgare*, *Quercus* *brantii*, and *Falcaria* *vulgaris* could increase the *Bcl*‐*2* expression level. Ethanol‐induced cellular damage downregulates this gene. Considering the protective effects of the extracts on the gastric ulcer and the mechanisms involved, it can be stated that *Bcl*‐*2* is upregulated due to the effective substances present in the extracts of the herbs mentioned above.

## 5 CONCLUSION

The hydroalcoholic extracts of *Quercus* *brantii* and *Falcaria* *vulgaris* can significantly protect the gastric mucosa against ethanol‐induced damage. Such protection was shown to a large extent in the form of a decrease in the gastric ulceration index and reduction or inhibition of edema. The results of the present study indicate the efficacy of the hydroalcoholic extracts of *Quercus* *brantii*, *Falcaria* *vulgaris*, and *Cirsium* *vulgare* (especially *Quercus* *brantii*) in improving the damage caused by free radicals and exerting protective effects on the EIGU model. Furthermore, these effects prevented the use of antioxidant sources, maintained MDA at normal levels, brought the levels of inflammatory factors back to their normal levels, and regulated the expression levels of the genes. In the present study, the evaluation of the expression levels of *C*‐*fos*, *C*‐*myc*, *caspase*‐*9*, and *Bcl*‐*2* genes revealed that the extracts had regulatory effects on gene expressions. Hence, and due to the anti‐gastric ulcer effects of the extracts, the expressed levels of *C*‐*fos*, *C*‐*myc* (as a regulator of cell proliferation and differentiation), and *caspase*‐*9* (as an apoptotic regulator) decreased. The expression level of *Bcl*‐*2*, an anti‐apoptotic gene, increased. These changes occurred because of the gastric ulcer improvement induced by the use of extracts. According to the results of previous studies and those of the present study, among the effective components of the herbal extracts examined in the present research, flavonoids may be the main effective factor in these herbs.

## CONFLICT OF INTEREST

There is no conflict of interest to disclose.

## AUTHORS’ CONTRIBUTION

Ali Mohammad Basatinya, Javad Sajedianfard, Kaveh Rahimi, Abolfazl Farahi, and Mahbobeh Kamrani Mehni collected the data, designed the project, and performed the statistical analysis. Saeed Nazifi accomplished serum test analysis. Saied Hosseinzadeh completed RT‐PCR assay. Ali Mohammad Basatinya, Amin Derakhshanfar, and Sina Salavati designed figures and drafted the initial manuscript. Ali Mohammad Basatinya, Javad Sajedianfard, and Saied Hosseinzadeh reviewed and revised the final version of the manuscript. All authors revised and approved the final submission and agreed on all aspects of this work.
